# Common ground type five level inverter with voltage boosting for PV applications

**DOI:** 10.1038/s41598-022-09008-z

**Published:** 2022-03-22

**Authors:** M. Jagabar Sathik, Dhafer J. Almakhles

**Affiliations:** Renewable Energy Lab, College of Engineering, Prince Sultan University, Riyadh, Saudi Arabia

**Keywords:** Electrical and electronic engineering, Photovoltaics

## Abstract

The boost-switched capacitor inverter topology with reduced leakage current is highly suitable for distributed photovoltaic power generation with a transformerless structure. This paper presents a single-stage 5-level (5L) transformerless inverter with common ground (CG) topology for single-phase grid-connected photovoltaic application. A generalized version of the proposed topology is also presented. The proposed topologies are derived by combining the dc/dc boost converter and switched capacitor cell. The primary focus has been given to the 5L version of the proposed topology. The number of switch counts is reduced, the maximum voltage gain is four times, and the inrush current is suppressed due to the input inductor configuration. Also, the voltages across the switched capacitors (SCs) are self-balanced. The negative source terminal and the load are connected directly to suppress the leakage current. It is thoroughly compared with other recent CG-Type topologies to attest to the advantages of the proposed topology. The laboratory prototype is developed for 600 W with the maximum efficiency is 94.21%, and the maximum source current is not more than 25A. Further, the operation of the proposed topology is verified under dynamic loading conditions, and the results are presented.

## Introduction

Multilevel inverters are well-matured power converters, and they are widely used in various applications, including renewable energy sources, AC drive, HVDC, etc.,^1,2^. However, the number of dc sources and voltage boosting is another big challenge in conventional MLIs. To increase the output voltage, several single dc sources switched capacitor multilevel inverter (SCMLI) topologies were reported^3,4^. In this SCMLI, topologies are suitable for PV applications, but the leakage current is another challenge in SCMLI topologies. The transformer-less inverter is a quite attractive power converter for PV applications because it doesn’t require a low-frequency, bulky and costly transformer (as the name suggests) for grid interface. The absence of a transformer leads to leakage current due to the non-existence of galvanic isolation. Here, the leakage current reduction can be achieved by adding additional power components to the inverter^5,6^. Several approaches like ac decoupling, dc decoupling, H-Bridge zero voltage rectifier (HB-ZVR), and midpoint clamped methods are available^7^ for addressing the leakage current issue. Although these approaches successfully suppress the leakage current to some extent, they fail to eliminate it. This issue is effectively addressed by the common ground (CG) type topologies introduced in^8^ by directly connecting the negative terminal of PV and the grid's neutral terminal, leading to zero leakage current. The CG type inverters often use a virtual dc source which can be either a floating capacitor (FC) or a switched capacitor (SC)^6^. In^9,10^, the topology uses a floating capacitor which requires high capacitance values to maintain the voltage across the FC^11^. In order to avoid the high capacitance value, a self-balancing topology is proposed in^12^. However, this topology uses one SC and multiple dc-link capacitors. The SC voltage is equal to the sum of all the dc-link capacitors, which increases the SC's voltage rating.

Further, the direct charging of SC with a voltage source will introduce a high capacitor charging current which requires devices of a higher current rating. To overcome the above issue, a soft charging method is proposed in a few SC topologies by inserting an inductor in the charging path^12–15^. However, the current is not suppressed dramatically due to the soft charging inductor's low inductance value (in (µH). To further suppress the charging current, the inductance should be increased to mH range, but this is not suitable because the high inductor value will disturb the nature of inverter operation. Hence, the topology should be designed so that the use of an inductor with high inductance should create any disturbance in the topology. In^16–18^, the inductor is used in the circuit, and its dual playing role is to (i) act as soft charging and (b) energy storage. However, the voltage of the topology presented in^16–18^ is four times that of the *v*_*in,*_ and it has more number of switches, and the voltage stress on the switches is twice the output voltage. Further, a five-level inverter topology is proposed in^16^ to reduce the voltage stress. In this topology, the number of device counts is high, and the voltage gain is four times that of the *v*_*in,*_ but the switch count is not reduced.It is important to mention that both the proposed topology and the one in^16^ fall under a common ground type inverter category. However, the proposed topology in this work has a lower number of switching devices compared to the topology in^16^. While the proposed topology has SEVEN switching devices, the topology in^16^ has TEN switching devices.Consequently, the proposed topology has a lower number of ON State switches, gate driver circuits, and other supporting components like heat sinks. This paper proposes a five-level CG type transformerless inverter topology with reduced switch count and high voltage boosting capability. The output voltage (*v*_*o*_) is four times (Quadratic Boost Inverter—QBI) the input voltage (*v*_*in*_). The other merits of this topology are given below:

i. The required number of IGBT is less than^14–18^.
ii. Suppression of inrush and leakage current.
iii. Suitable to operate in high reactive power load.
iv. The maximum voltage stress on the switch is equal to the *v*_*o*_ compared to^18^.
v. The maximum voltage stress across the SC is 2*v*_*in*_.

### Proposed 5L-QBI topology

#### Description of proposed topology

**Figure 1:**
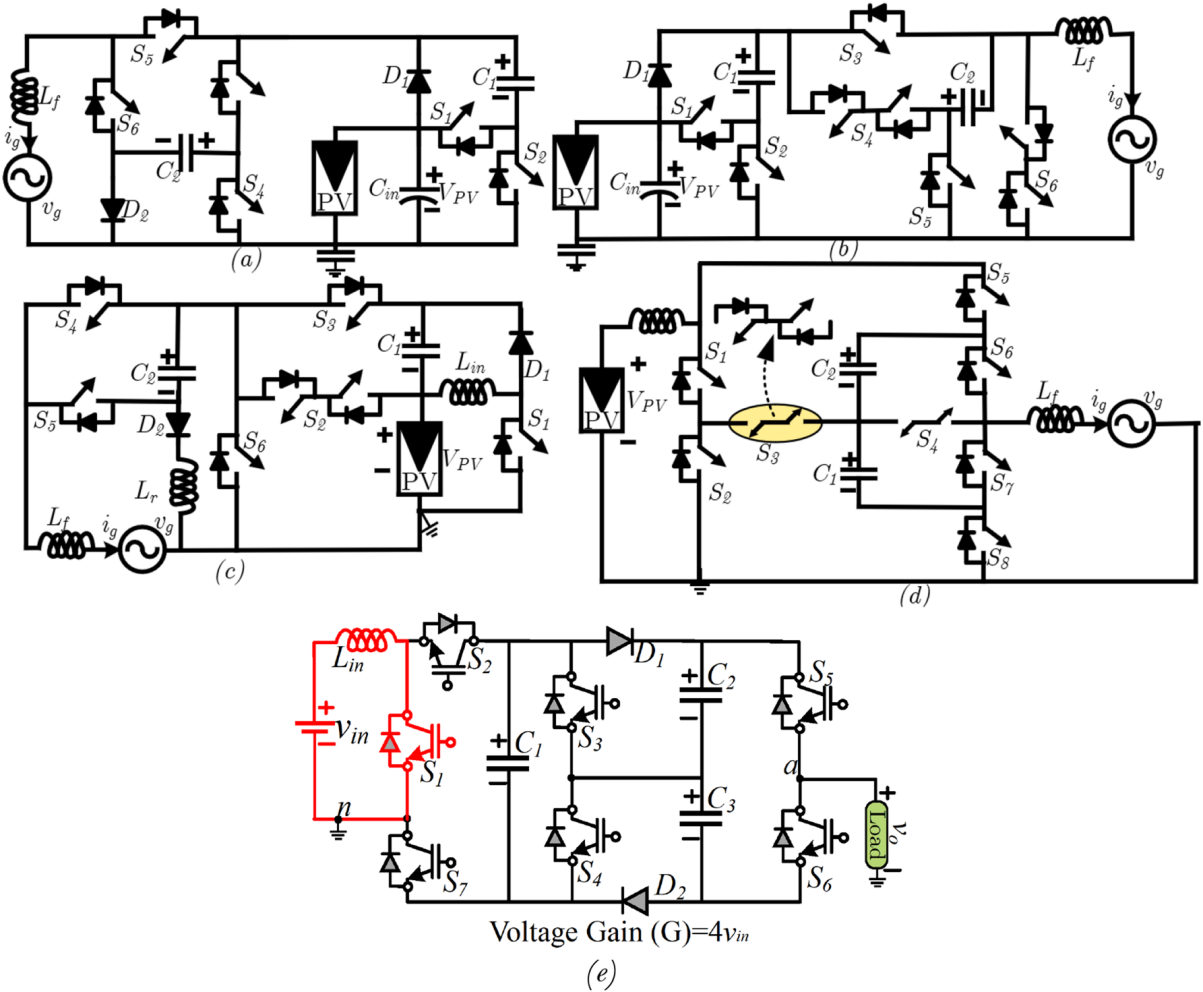
Various common ground 5L inverter circuit diagram (**a**) presented in^14^, (**b**) presented in^15^, (**c**) presented in^17^, (**d**) presented in^18^ and (**e**) proposed 5L-QBT topology.

The circuit diagram of the proposed single-stage topology is shown in Fig. 1. The proposed topology uses seven switches, two diodes, and three capacitors. Each capacitor is charged to 2vin, and the switch S1 is operated in a 50% duty cycle.

#### Modes of operations

**Figure 2:**
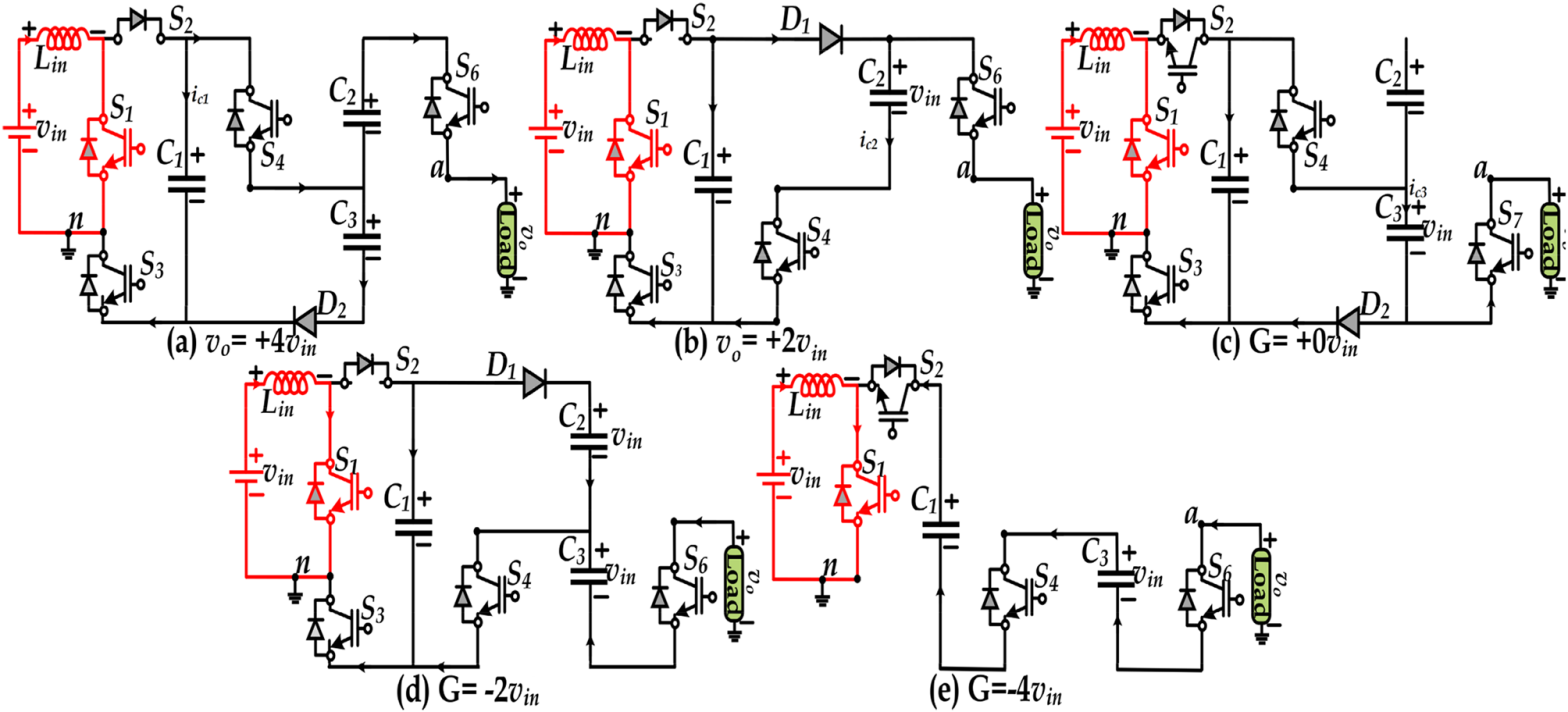
Modes of operation (**a**) L_+2_ (+ 4 *v*_*in*_), (**b**) L_+1_ (+ 2 *v*_*in*_), (**c**) L_0_ (+ 0 *v*_*in*_), (**d**) L_−1_ (−2 *v*_*in*_), and (**e**) L_−2_ (−4 *v*_*in*_).

The proposed 5L-QBI inverter topology simultaneously performs both boosting and inverting operations. For better understanding, Fig. 1 is considered for describing the modes of operation. In each mode, the inductor charging and discharging are explained with corresponding circuit diagrams as depicted in Fig. 2a–e. Moreover, the duty cycle of the embedded boost converter is fixed to 50%, and the corresponding switching sequence is given in Table 1. The capacitors' charging currents are defined as i_c1_, i_c2_, i_c3_, and the load current io.

**Table. 1:** Switching sequence of 5L-QBI topology.

Level	ON state switches		*v*_*o*_	L_in_	C_1_	C_2_	C_3_
	S_2_-S_7_	S_1_					
L_+2_	011010	P_ON_	+ 4*v*_*in*_	Charging	↓	↓	↑
		P_OFF_		Discharging			
L_+1_	010110	P_ON_	+ 2*v*_*in*_	Charging	↑	↑	-
		P_OFF_		Discharging			
L_+0_	011001	P_ON_	0 *v*_*in*_	Charging	-	↑ or -	-or ↑
		P_OFF_		Discharging			
L_-1_	010101	P_ON_	− 2*v*_*in*_	Charging	↑	-	↑
		P_OFF_		Discharging			
L_-2_	100101	1	− 4*v*_*in*_	Charging	↓	↑	↓

*D-Diode, P-Pulse, ↑-Charging ↓-discharging, P_ON/OFF_- Pulse ON /OFF.

#### Top positive voltage level (+ 4 v_in_)

In this mode, *v*_*o*_ is the sum of the voltage across the inductor and the capacitor C_1_ i.e., $v_{o} = v_{L} + v_{c1}$ and the $v_{L} = \left[ {{1 \mathord{\left/ {\vphantom {1 {(1 - d)}}} \right. \kern-\nulldelimiterspace} {(1 - d)}}} \right]v_{in}$, where the *d* is kept at a 50% constant value. Here, the C_1_ is discharging, and C_2_ is charging to *2 v*_*in*_*.* The switches S_3_ and S_6_ are turned ON, and the anti-parallel diode of S_2_ and diodes D_2_ and D_a_ are conducting. When the dc source is connected parallel to the SCs, the SCs draw a considerable current during starting, often called the inrush current. The inrush current magnitude is too high, and it can be suppressed by adding more resistance or inserting the inductor in the charging path. It can be observed that the proposed 5L-QBI topology uses an inductor and it serves two purposes which are as follows: -(i) boosting the input voltage and (ii) limit/suppressing the inrush current. Note that the proposed topology belongs to the boosting inverters family with 4 gain i.e., the input current on the input side is 4-times higher than the output current. Due to the inductor on the input side, the inrush current in the proposed topology is reduced by 5-times as compared to the non-inductor topology. Further, similar inductor-based SC-MLI topologies are already addressed^16–18^.

*Switch Stress:* The maximum voltage (MVS) and current stress (MCS) for each level are 2 *v*_*in*_ and *i*_*c2*_+*i*_*o,*_ respectively.

#### First positive voltage level (+ 2 v_in_)

The value of *v*_*o*_ is two times of *v*_*in*_ due to the storage element inductor is acting as a voltage booster. The capacitor C_1_ gets charged to this level, but C_2_ is neither connected with the source nor load. The switch S_4_ is turned ON to charge the upper capacitor C_1_ and the switches in ON state are S_4_, S_5_, and S_6_. *Switch Stress:* The MVS and MCS are *v*_*in*_ and $i_{c1} + i_{o}$.

#### Zero voltage level (+ 0 v_in_)

The freewheeling path for the load is provided in this mode. The load voltage equals zero, but the capacitor C_2_ is charged via S_3_, S_7,_ and D. The switch S_1_ continues to act as a boost converter switch at a fixed 50% of the duty cycle. The voltage across C_2_ is equal to 2*v*_*in*_. *Switch Stress:* The MVS and MCS are respectively 2*v*_*in*_ and $i_{c1} + i_{o}$.

#### First negative voltage level (-2 v_in_)

The SC C_2_ discharges to the load via D_a_, S_4_ & S_7,_ and the upper SC C_1_ is charged to 2*v*_*in*_ simultaneously. The switch S_1,_ which is turned ON and OFF at high frequency, forms the boost converter and the inductor L_in_ and capacitor C_1_. *Switch Stress:* The MVS and MCS are respectively *v*_*in*_ and *i*_*o*_.

#### Top negative voltage level (-2 v_in_)

In this mode, the switch S_1_ is turned ON to provide the path for the 5th voltage level, as shown in Fig. 2e. Here, the **switch** S_1_ will not operate as a boost converter switch, and the pulses change over to the inverter pulses.

Now, the load voltage is equal to sum of the *C*_*2*_ & FC i.e. *v*_*o*_ = -3*v*_*in*_*/2.* The Diode *Dx* provides the current path during the lagging or leading power factor*.* The above discussion clearly shows that the proposed topology uses fewer ON state switches in each voltage level. The stress analysis on the switches is the important parameter for capacitor self-balanced inverter topologies. The high inrush current will occur during the parallel connection of FC and *v*_*in*_. The current small inductor can be added to the circuit loop to prevent the inrush. It confirms that the proposed topology's maximum voltage stress equals 2*v*_*in*_ and current stress is *i*_*o*_ + *i*_*c*_ occurred in only two switches. Other topologies presented in^8–11^ needed four switches with high current stress.

#### Extended structure

**Figure 3:**
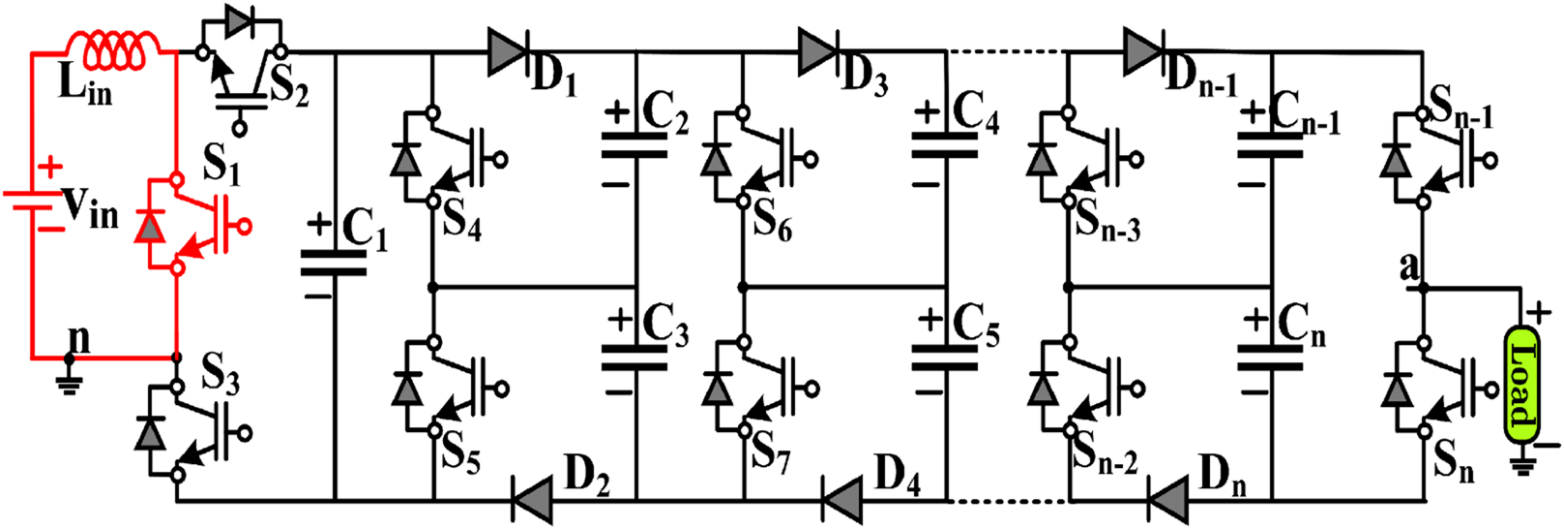
Proposed extended structure.

Figure 3 shows the extended structure of the proposed topology. The boosting module is connected parallel to the dc source, and it can be extended to the “*n*” number of modules. Each module consists of two capacitors, two diodes, and two switches. Further, all the capacitors are charged to $v_{in} /(1 - d)$ , i.e. $2v_{in}$ where $d = 0.5$ constant.

The number of output voltage level (*N*_*Level*_), switches (*N*_*Switch*_), diode (*N*_*Diod*e_) and capacitors (*N*_*Capacitors*_) for “*n*” number of modules is given in (1)-(4).

1 $$ N_{Level} = 2n + 3 $$

2 $$ N_{Switch} = \, 2n + 5 $$

3 $$ N_{Diode} = 2n $$

4 $$ N_{Capacitors} = 2n + 1 $$

5 $$ Output \, Voltage \, Gain \, \left( {v_{G} } \right) = 2\left( {n + 1} \right)v_{in} $$

The voltage gain of the extended topology increases with the increase in the number of modules as given in (5).

### Modulation technique and passive component design guidelines

#### Proposed PWM Scheme

**Figure 4:**
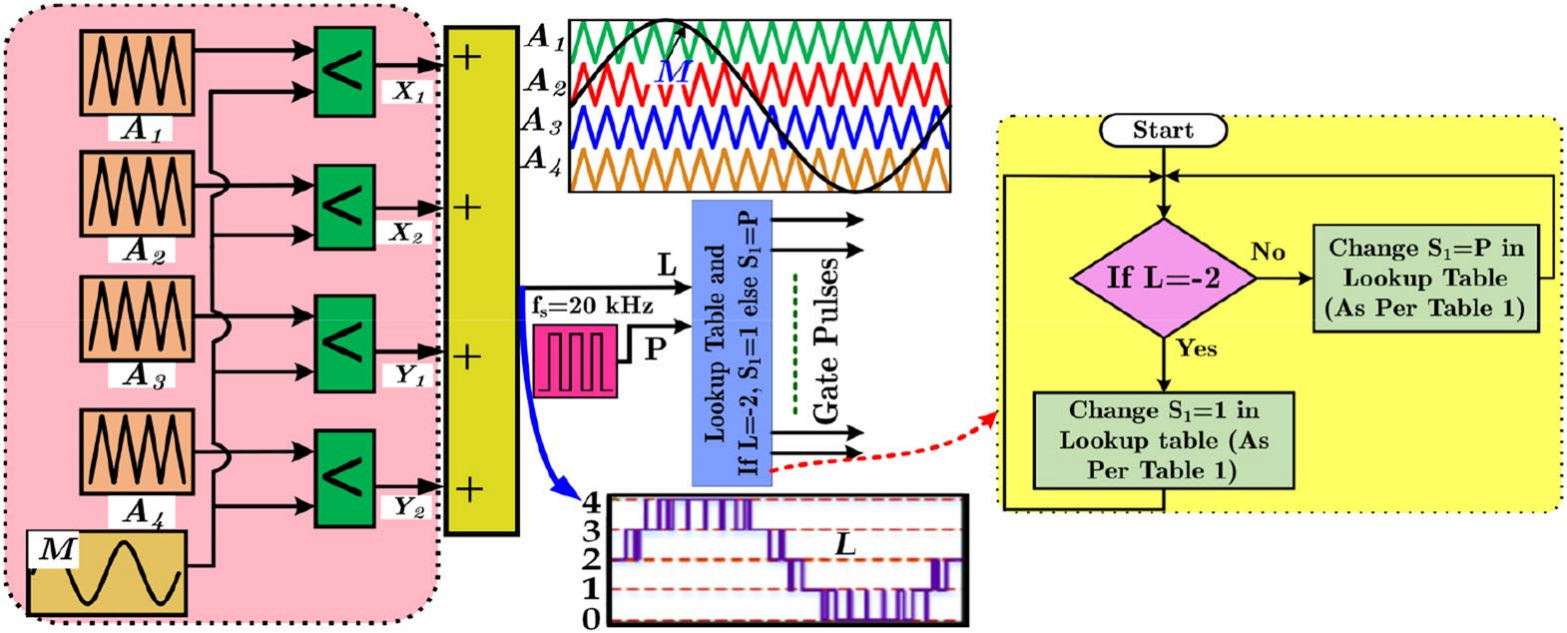
Proposed PWM scheme.

The proposed PWM technique is depicted in Fig. 4. The level-shifted triangular carrier signal (A_1_-A_4_) is used in the conventional sinusoidal PWM technique. The switching sequence for each voltage level is stored as given in Table 1. The parameter “L” is the sum of all the comparators’ output. L is given as the input to the lookup table. Except level -2, the switch S_1_ is operated by an independent pulse (ON/OFF) train with a switching frequency of *f*_*s1,*_ as depicted in Fig. 4. When *L* is equal to the -2, S_1_ will be turned ON as Table 1. The front side of both inverters looks the same. However, the proposed topology has distinct features in which the pulse generation and switching schemes are not the same. Moreover, the proposed topology is extendable to N_Level_, whereas presented in^19^ is limited to nine-level. In^19^, a constant is compared with the carrier to generate pulses that are used to control the switches (S_1_ and S_2_). However, the magnitude of this constant is not clear and makes it doubtable to be used for the MPPT, whereas the proposed topology is driven by an independent pulse generation scheme that makes it an optimal option to achieve the MPPT. The logic sequence for switches is given in (10).

6-9 $$ \left. \begin{gathered} i_{L} = \frac{1}{L}\int {\left\{ {\left( {1 - S_{1} } \right)\left[ {v_{C3} \left( {L_{ + 2} + L_{ + 0} } \right) + v_{C2} \left( {L_{ + 1} + L_{ - 1} } \right)} \right] + S_{1} v_{in} } \right\}dt} \hfill \\ v_{C1} = \frac{1}{{C_{1} }}\int {\left\{ {\left( {L_{ + 2} + L_{ + 0} } \right)\left[ {\left( {1 - S_{1} } \right)i_{L} - \left( {v_{C1} - v_{C3} } \right)/r} \right] + \left( {L_{ + 1} + L_{ - 1} } \right)\left[ {\left( {1 - S_{1} } \right)i_{L} - \left( {v_{C1} - v_{C2} } \right)/r} \right] + L_{ - 2} i_{Load} } \right\}dt} \hfill \\ v_{C2} = \frac{1}{{C_{2} }}\int {\left\{ {\left( {L_{ + 2} + L_{ + 0} } \right)\left[ { - i_{Load} } \right] + \left( {L_{ + 1} + L_{ - 1} } \right)\left[ {\left( {v_{C1} - v_{C2} } \right)/r - i_{Load} } \right]} \right\}dt} \hfill \\ v_{C3} = \frac{1}{{C_{3} }}\int {\left\{ {\left( {L_{ + 1} + L_{ - 1} } \right)\left[ { - i_{Load} } \right] + \left( {L_{ + 2} + L_{ + 0} } \right)\left[ {\left( {v_{C1} - v_{C3} } \right)/r - i_{Load} } \right]} \right\}dt} \hfill \\ \end{gathered} \right\} $$

10 $$ \begin{gathered} \begin{array}{*{20}l} {\left. \begin{gathered} S_{1} { = }P{\text{ for }}L \ne - 2 \hfill \\ { = }1 \, L = - 2 \hfill \\ \end{gathered} \right\}} \hfill \\ {S_{2} = Y_{2} , \, S_{3} = X_{2} + Zero, \, S_{4} = X_{1} \overline{{X_{2} }} + Y_{1} \overline{{Y_{2} }} ,} \hfill \\ \end{array} \hfill \\ \hfill \\ S_{5} = Y_{2} , \, S_{6} = X_{1} , \, S_{7} = Y_{1} \hfill \\ \end{gathered} $$

#### Design of L and C components

**Figure 5:**
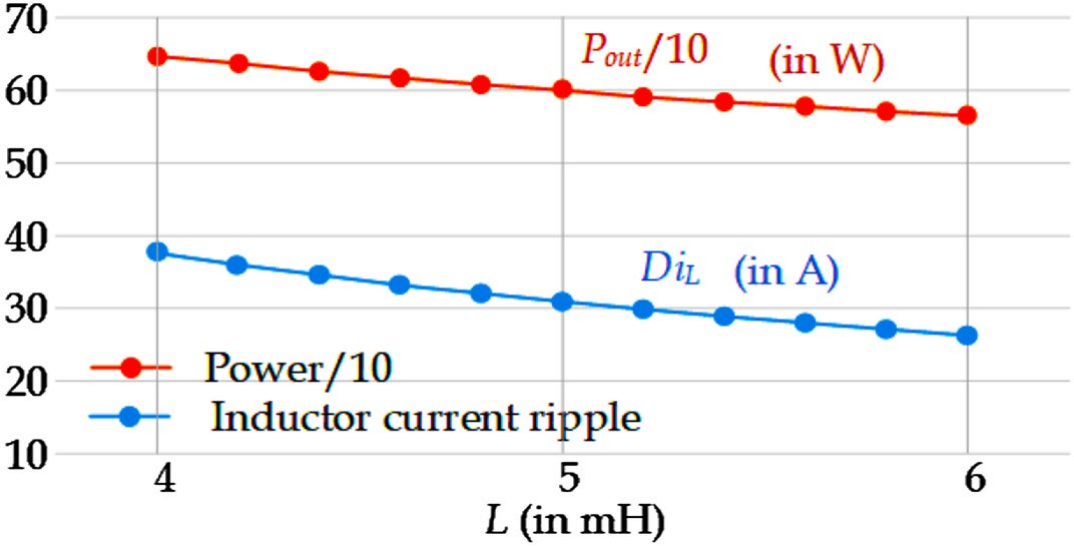
The inductor current ripple and output power vs the value of inductance for *L*_*in*_.

**Figure 6:**
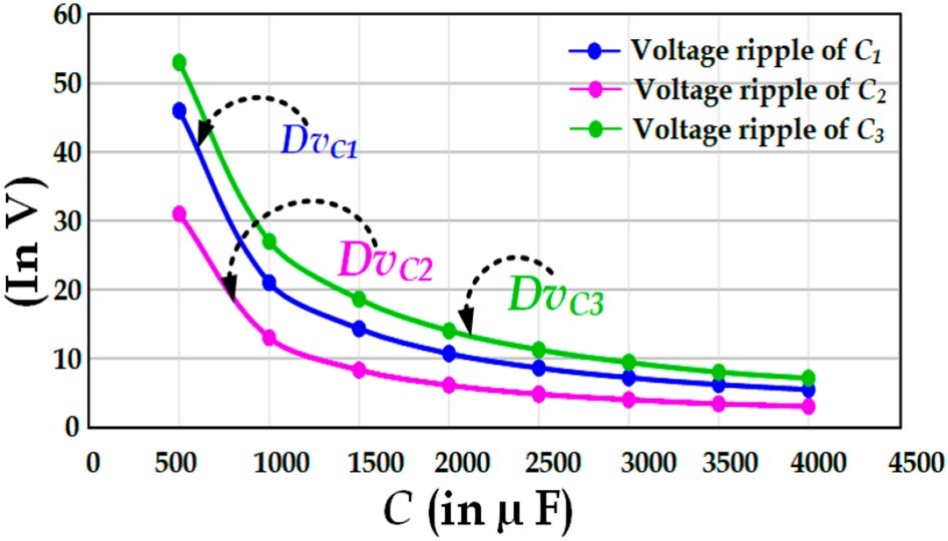
The capacitor voltage ripple vs the value of capacitance for the capacitors *C*_*1*_, *C*_*2*_, and *C*_*3*_.

The proposed topology switching function analysis is used to design the capacitor and the inductor. The following switching functions *L*_*k*_ (where *k* ∈ {2,1, 0, -1, -2}) and *S*_*1*_ are defined. The switching function *L*_*k*_ assumes the value ‘1’ if the generated voltage level is ‘*k*’. However, if the above condition is not satisfied, *L*_*k*_ is equal to ‘0’. The other switching function *S*_*1*_ = 1, when switch *S*_*1*_ is turned on, otherwise *S*_*1*_ = 0. The following equations may be derived for the current of inductor *L*_*in*_ (*i*_*L*_) and the voltages of capacitors *C*_*1*_, *C*_*2*_, *C*_*3*_ (respectively *v*_*C1*_, *v*_*C2*_, and *v*_*C3*_). The (6)-(9) *i*_*Load*_ is the load current, and *r* is the charging path resistance. By solving these equations using numerical methods for an R-L type of load with R = 50 ∧, L = 1mH, r = 100 m∧, and *v*_*in*_ = 50 V at 0.95 modulation index (m_a_ = 0.95), a few graphs are obtained as given in Figs. 5 and 6. Figure 5. shows that the inductor current ripple varies with the inductance *L*_*in*_. However, the output power is also slightly influenced by the inductance variation, as observed from Fig. 5. From the curve given in Fig. 5, the inductance value of *L*_*in*_ is chosen as 3 mH which corresponds to 600 W and 30 A current ripple.

Figure 6. shows the capacitor voltage ripple vs capacitance for the capacitors C_1_, C_2_, and C_3_. It may be observed that C_3_ has a slightly higher voltage ripple than C_1_ or C_2_ whereas C_2_ has the lowest voltage ripple. It is worth noting that this curve is plotted by considering *C*_*1*_ = *C*_*2*_ = *C*_*3*_ = *C*.

**Figure 7:**
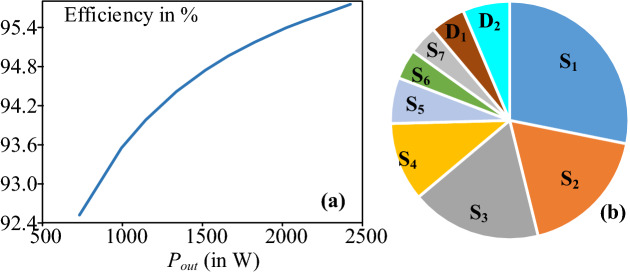
**(a)** Efficiency vs the output power curve and (**b**) loss distribution considering various power switches at 2.4 kW. (PLECS V4.1.2- https://www.plexim.com/).

*Loss analysis:* Loss analysis of the proposed topology is carried out in PLECS software with the following devices models: IKW30N60T_IGBT and IKW30N60T_Diode. The loss analysis is carried out with the following parameters: *V*_*in*_ = 50 V, L_in_ = 5 mH, C_1_ = C_2_ = C_3_ = 3000 µF, and *f*_*s*_ = 5 kHz. The efficiency vs output power curve is shown in Fig. 7a where a drop in efficiency is observed at lower output power. The loss distribution for various switches in the proposed topology is shown in Fig. 7b for 2.4 kW. It may be observed that the losses for switch S_1_ is higher than the other devices. In fact maximum amount loss of the system is shared by the switches S_1_, S_2_, S_3_, and S_4_.

## Results and discussions

**Figure 8:**
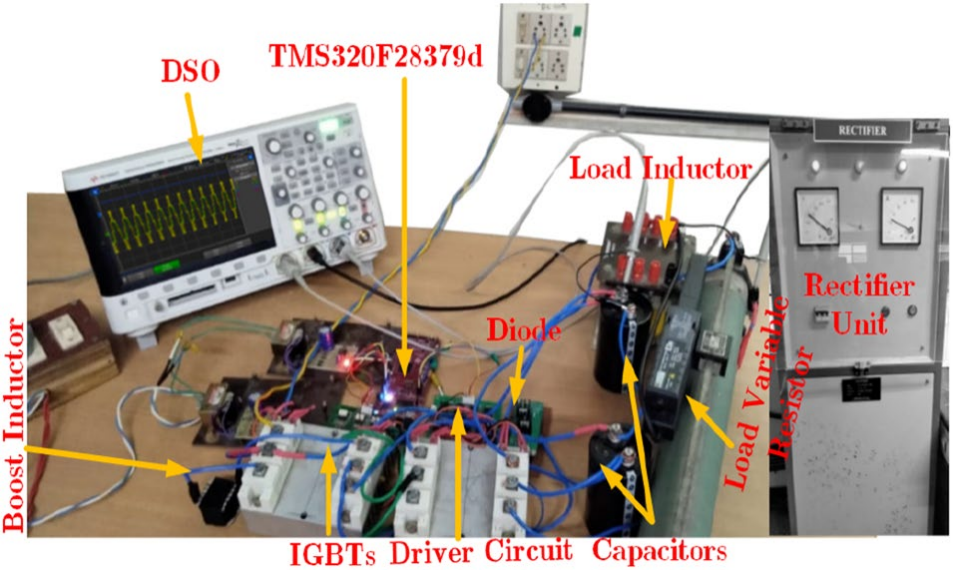
Prototype Hardware Setup.

**Figure 9:**
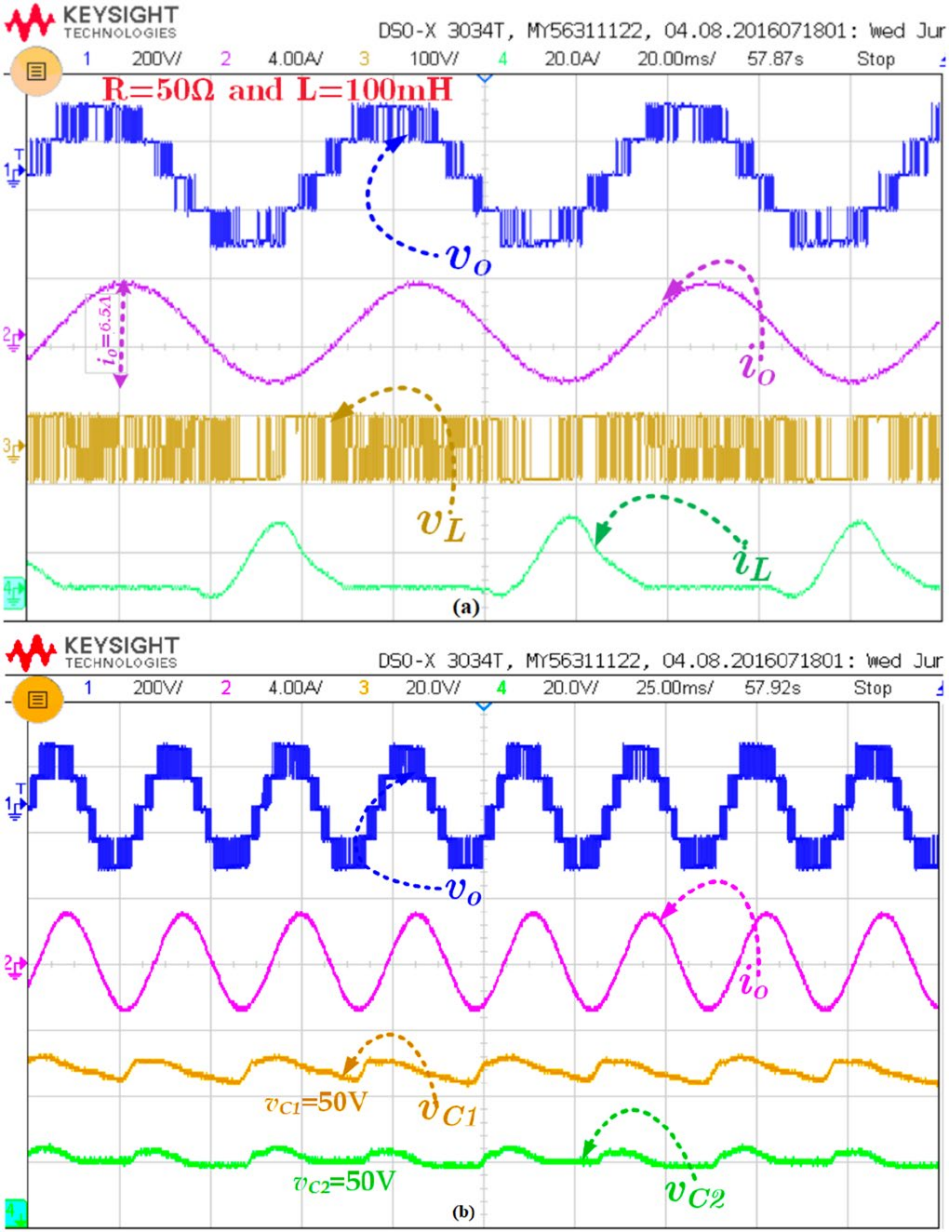
Experimental result of (a) *v*_*o*_*, i*_*o*_*, v*_*L*_*,* and *i*_*L*_ for R = 50Ω and L = 100 mH and (b) *v*_*o*_*, i*_*o*_*, v*_*C1*_*,* and *v*_*C2*_*.*

**Figure 10:**
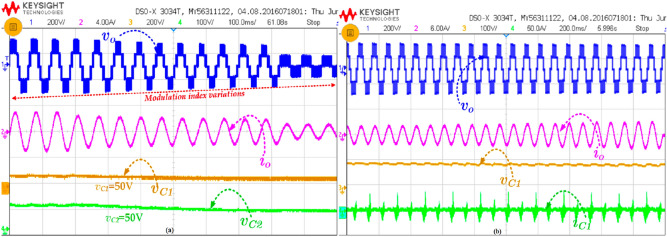
Experimental result of (**a**) modulation index variations and (**b**) load variations.

The prototype hardware setup of proposed topology is shown in Fig. 8. The Semikron IGBT 600 V/75A is used in this circuit. Further, the TLP 250 driver circuits is used with external RC delay circuit to provide the deadtime of 3 µs—4 µs for each switch to avoid the short circuit. Moreover, the 3 mH iron core inductor is used, and 3000 (F capacitance capacitors are used. Initially, the input voltage is given to the circuit and the switch S_1_ is turned on/off to boost the voltage. The pulses are generated from the MATLAB and embedded into the texas controller TMS320F28379D. The Keysight DSOX2002A probe is used to measure the voltage and probe can withstand up to 300Vrms and there was a small voltage fluctuation during the experimental measurement. Since the output power is low, the waveforms are not affected.. Figure 9a shows the measured value of output voltage, current and inductor current during the steady-state condition for the RL load (50Ω + 100mH) @ power factor of 0.85 lagging. The maximum output voltage (*v*_*o,max*_) is 200 V, maximum output current (*i*_*o,max*_) = 3.3 A. Further, the voltage across the inductor is maintained at 100 V and the maximum current is 25 A as shown in Fig. 9b. Moreover, the capacitor across the voltage C_1_ and C_2_ is captured and shown in Fig. 9b. Further to investigate the proposed topology is dynamic performances, the modulation index variation and load variations are applied and results are measured as shown in Fig. 10a and b. The modulation index is reduced from the 1.0 to 0.45 by varying the potentiometer. From Fig. 10, it is confirmed that the voltages of the capacitors are slightly reducing during the low modulation index. This is because the duration of top negative level is reduced, where the inductor gets continuously charged. However, the capacitors are discharged during the negative cycle.

**Figure 11:**
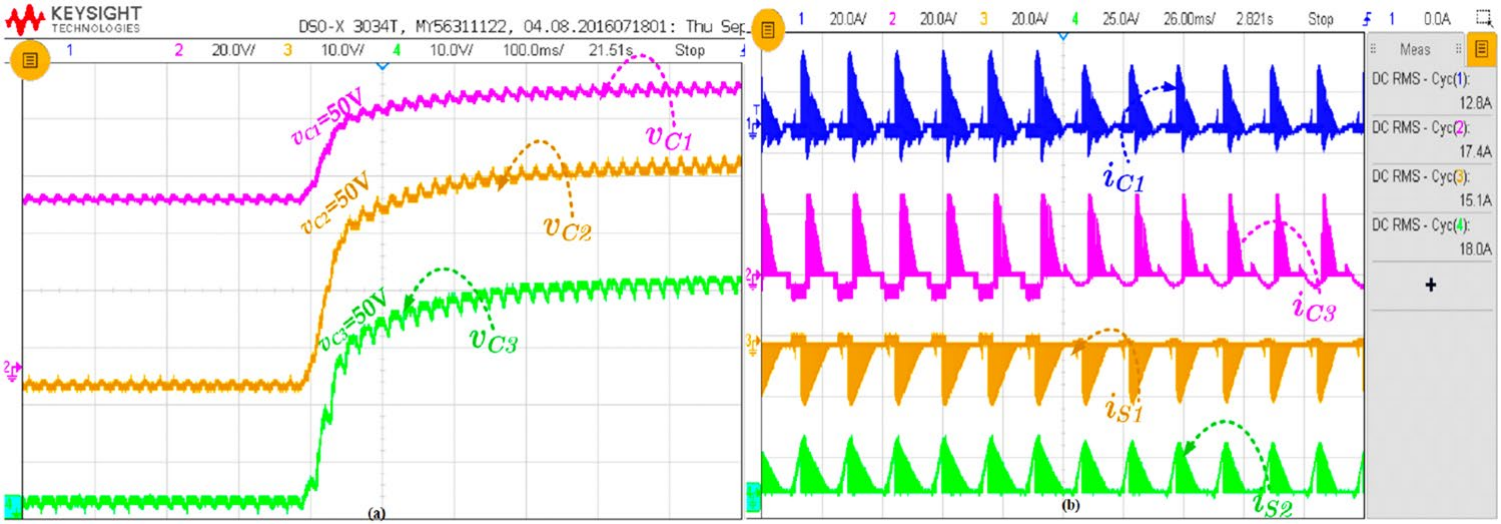
Experimental result of (**a**) capacitors voltages and (**b**) voltage and current of capacitors C_1_ and switch S_1_.

Most of the inverter loads are highly dynamic in behaviour. Therefore, a highly reliable topology is needed to adopt the load variations. The proposed topology is verified under the varying load condition, and the corresponding results are shown in Fig. 11. In fact, the load variations are carried out by keeping the load inductance constant but varying the resistance of the rheostat.

The capacitor (C_1_) current and voltages are measured during this load variation, as shown in Fig. 11a. The capacitor voltage from the initial duration is shown in Fig. 11a. This waveform is captured by discharging all the capacitors used in the circuit. In fact, the capacitor charging is too high due to the low resistance series to the capacitors. The various capacitors and their currents and switch currents are shown in Fig. 11b. Without the inductor, the charging will be ten times higher than the load current, but the inductor, which acts as a current limiter, limits the maximum charging current.

**Figure 12:**
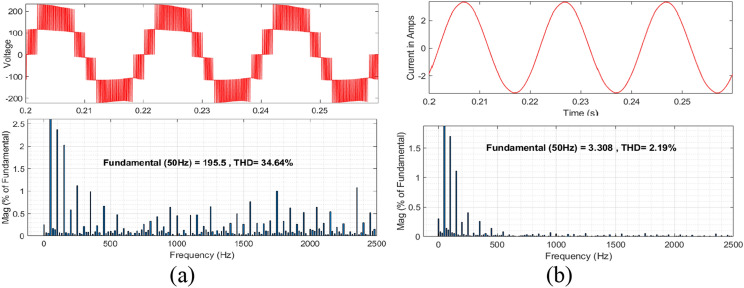
Total harmonic distortion (**a**) for Voltage and (**b**) for current.

**Figure 13:**
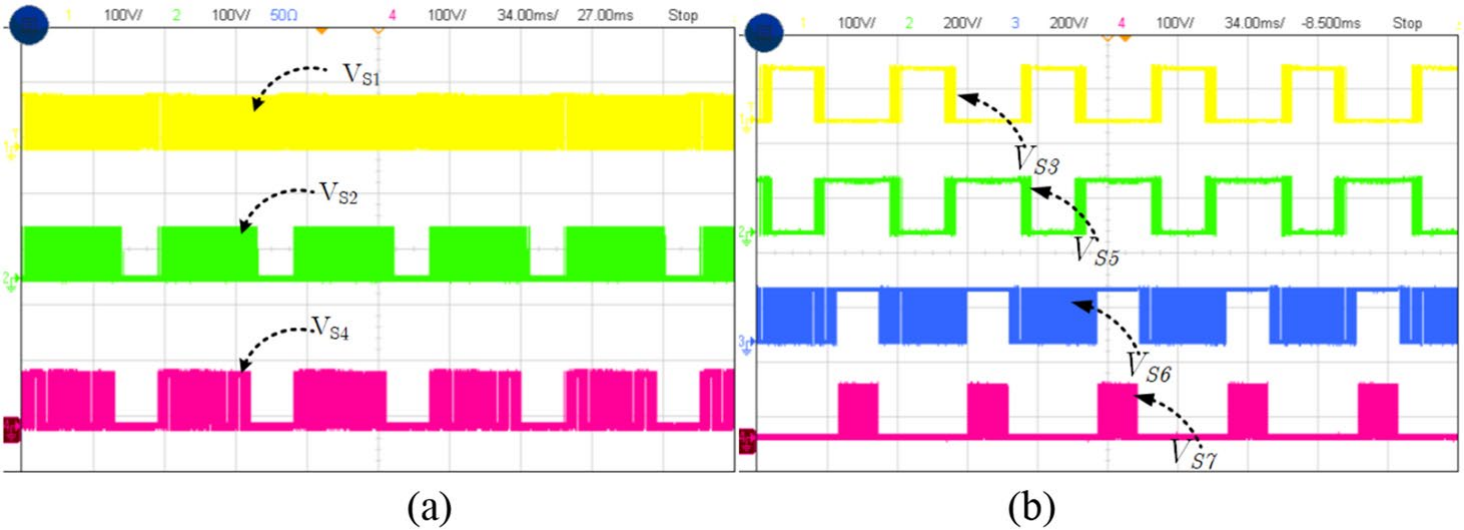
Voltage across the switches (**a**) V_S1_, V_S2_, V_S4_ and (**b**) V_S1_, V_S2_, V_S4_.

The summary for the comparison of recent switched capacitor and common ground type topologies are listed in Table 2. In this^16^, uses the ten switches whereas the proposed topology uses seven switches with additional two diodes. It is worth mentioning that the diode is lower than the switches and doesn’t require gate driver circuits. Obviously, the cost of the proposed topology is less than the^16^ and achieves better performance. Another CG-type topology^17,18^ topologies generate the five-level with boosting factor 2 M/(1-D). However, these topologies' switch count is high but in^17^, the voltage gain is half of the proposed topology. Further, the series connection of dc source and capacitors will introduce the pulsating dc which affect the source. The simulated total harmonics distortion (THD) for the proposed topology is shown in Fig. 12 without output filter. The voltage THD is 34.64% and for current THD 2.19%. Finally, the voltage across the switches (V_S1_-V_S7_) is given in Fig. 13 and its confirming that the maximum blocking voltage is not more than output voltage (*v*_*o*_).

**Table. 2:** Summary Comparison.

Ref	N_L_	N_S+DC_	N_D_	N_SC_	T_C_	v_SC_	G	I_CC_	Efficiency (%)
^8^	5	8 + 8	–	1	17	v_in_	M	No	97
^9^	7	8 + 8	1	2	19	2v_in_	M	No	96.3
^14^	5	6 + 6	2	2	16	2v_in_	2v_in_	No	98.1
^15^	5	8 + 6	1	2	17	2v_in_	2v_in_	No	96
^16^	5	10 + 10	–	2	22	2v_in_	2 M/(1-D)	Yes	97.13
^17^	5	7 + 6	2	2	17	2v_in_	M/(1-D)	Yes	97
^18^	5	10 + 8	–	2	20	2v_in_	2 M/(1-D)	Yes	98.2
Prop	5	7 + 7	2	3	19	2v_in_	2 M/(1-D)	Yes	94.5

N_L_-Number of level, N_S+DC_-Number of switch and driver circuits, N_D_-number of diodes, N_SC_-Number of switched capacitor and T_C_-total power component, V_SC_-voltage rating of SC, G-voltage gain and I_CC_-Input continuous current.

## Conclusion

A new five-level inverter topology with the common ground was discussed. The detailed operation of each mode and PWM scheme was presented. The integrated boost inverter is operated so that it suppresses the SC charging current and boosts the input voltage. The extended structure of the proposed topology was also discussed. Since the 5L-QBI is a common ground type, the leakage current is eliminated, allowing the proposed topology to be more suitable for transformerless applications. The main advantage of the proposed topology is the availability of the charging state for every switching state except the -2 level. Further, the number of devices and rating of the devices is less than the similar topologies. A measured efficiency value is 94.21% at ~ 600 W, which is close to the simulation value of 94.8%. From the above points, the proposed topology is a suitable candidate for transformerless PV application with reduced device count, reduced current stress, and voltage boosting capability.

## Data Availability

The datasets analyzed during the current study are available from the corresponding author on reasonable request.
